# YtrG is a functional part of the *ytrGABCDEF* operon and contributes to phenotypic heterogeneity in *Bacillus subtilis*

**DOI:** 10.1128/spectrum.02912-26

**Published:** 2026-10-06

**Authors:** Margareth Sidarta, Pauline Hammer úr Skúoy, Martin Benda, Jörg Stülke, Michaela Wenzel

**Affiliations:** 1 Division of Molecular Bioscience, Department of Life Sciences, Chalmers University of Technology 11248, Gothenburg, Sweden; 2 Centre for Antibiotic Resistance Research in Gothenburg (CARe) 606685, Gothenburg, Sweden; 3 Department of General Microbiology, Institute of Microbiology and Genetics, Georg-August University Göttingen 200972 , Göttingen, Germany; 4 Department of Parasitology, Faculty of Science, BIOCEV, Charles University 37740, Prague, Czech Republic; University at Albany, Albany, New York, USA

**Keywords:** ABC transporters, antimicrobial protein, fluorescence assays

## Abstract

The *Bacillus subtilis ytrGABCDEF* operon consists of the transcriptional repressor YtrA, the ATP-binding cassette transporter subunits YtrB, YtrC, YtrD, YtrE, and YtrF, likely forming two distinct transmembrane complexes (YtrB_2_CD and YtrE_2_F_2_), and the small monotopic transmembrane protein YtrG, which has so far remained uncharacterized. Overexpression of the operon leads to pronounced phenotypic heterogeneity characterized by a subpopulation of cells with strongly reduced length and width (“small cells”). Operon mutants that constitutively express *ytrG*, together with incomplete transporter variants, show pronounced lysis of these small cells that is not observed when the whole operon is overexpressed. Based on this observation, together with bioinformatic analyses of the YtrG protein sequence that revealed some structural features associated with membrane-active toxins, we investigated whether YtrG could function as a toxin underlying small cell lysis. Synthetic YtrG peptide displayed moderate antibacterial activity against *Bacillus subtilis* and *Escherichia coli*, but did not induce the characteristic small-cell or lysis phenotypes, and had only minor effects on the cell membrane. Likewise, overexpression of *ytrG* in the wild-type background did not impair growth and caused neither small cells nor cell lysis. In contrast, deletion of *ytrG* resulted in pronounced small-cell and lysis phenotypes that were partially complemented by ectopic *ytrG* expression, indicating that YtrG counteracts rather than promotes these effects. Together, our findings exclude YtrG as the cause of the small cell and lysis phenotypes, but identify it as an important factor required for small cell integrity.

**IMPORTANCE:**

The *Bacillus subtilis ytrGABCDEF* operon is activated by cold shock and cell wall-targeting antibiotics and has been established as a reliable marker for interference with lipid-linked cell wall precursor synthesis in antibiotic mode of action studies. Despite its highly specific induction pattern, the function of the operon remains debated. Recent studies point toward a role in balancing cell wall synthesis and autolysis, possibly related to cannibalism. Despite thorough genetic, functional, and structural characterization of the operon, the function of its smallest component, YtrG, has remained unaddressed. Our findings provide the first insights into the role of YtrG and show that it is indeed a functional component of the operon.

## INTRODUCTION

The *Bacillus subtilis ytrGABCDEF* operon consists of the transcriptional repressor YtrA, the ATP-binding subunits YtrB and YtrE, the transmembrane proteins YtrC and YtrD, and the extracytoplasmic substrate-binding lipoprotein YtrF (1). Originally proposed to form one ATP-binding cassette (ABC) transporter (YtrBCDEF) (2), newer data point toward two independent ABC transporters, YtrB_2_CD and YtrE_2_F_2_ (3). The structure of the YtrE_2_F_2_ complex has been confirmed with cryogenic electron microscopy (4). However, neither of these configurations account for YtrG, a small monotopic transmembrane protein of unknown function (1). The *ytr* operon has been implicated in a number of cellular functions including antibiotic and cold stress adaptation, cell wall synthesis, autolysis, sporulation, biofilm formation, motility, competence, and acetoin utilization (1, 2, 5, 6). While its precise function remains unclear, accumulating evidence points toward a role in balancing cell wall biosynthesis and autolysis (4, 5, 7).

We discovered that mutants overexpressing either the whole operon or parts thereof display a pronounced population heterogeneity characterized by a subpopulation of non-growing cells with considerably reduced cell length and width (“small cells”) (7). This phenotype was most pronounced during lag-to-exponential growth phase transitions and after mild temperature shocks from 37°C to 24°C. Interestingly, small cells were prone to lysis in strains that overexpress only part of the operon (Δ*ytrAB*, Δ*ytrAE*, Δ*ytrABE*, and Δ*ytrACD*) but did not lyse when the whole operon was overexpressed (Δ*ytrA*) (7). Taking together these observations with data from Yu et al., showing that extended incubation of cells overexpressing *ytrEF* results in microcolonies of survivor populations growing on lysed sibling colonies (4), we proposed that the small cell phenotype could be related to cannibalistic behavior, which is a common stress response strategy in *B. subtilis* (8). Thus, we speculated that non-growing small cells could represent lysis-resistant survivor populations that outlast their siblings and resume growth after the remaining cell population has lysed, making fresh nutrients available for renewed colony growth (7). In this scenario, the small cell lysis phenotype, observed in mutants expressing an incomplete operon, could possibly be attributed to an imbalance of cell wall synthesis and autolysis (7).

However, it is also possible that the small cell lysis behavior could be connected to the so far uncharacterized *ytrG* gene, which was not accounted for in our original mutant studies (6, 7). Thus, all strains that overexpressed an incomplete operon also force-expressed YtrG. As a single transmembrane-domain protein, YtrG bears some similarity to membrane-active toxins (9, 10). During strain construction, we observed that plasmid constructs containing the *ytrG* sequence were difficult to transform into our *Escherichia coli* cloning host. Hence, we hypothesized that *ytrG* may encode a membrane-active toxin. In this scenario, one or both ABC transporters could be involved in its secretion or membrane insertion. Overexpression of *ytrG* along with an incomplete transporter would then lead to its intracellular accumulation or faulty membrane incorporation, possibly causing the observed lysis phenotype. Curiously, when we tried to incorporate YtrG into the different possible transporter topologies in AlphaFold 3.0, we noticed that the program favored different subunit interactions in the presence and absence of YtrG (7). This observation led to an alternative hypothesis, namely that YtrG could possibly interact with one or both ABC transporters to modulate their function and promote cell lysis. To rule out these possibilities, we bioinformatically characterized the YtrG sequence, investigated the antibacterial effects of synthetic YtrG, and characterized *B. subtilis* mutants either lacking *ytrG* or expressing *ytrG* as the only gene of the operon. While we could indeed show mild antibacterial activity of YtrG, we could neither confirm a toxin nor a transporter-modulating function of the protein. Instead, YtrG appears to be involved in small cell integrity along with the other parts of the operon.

## RESULTS AND DISCUSSION

### Bioinformatic analysis of YtrG

**Fig 1:**
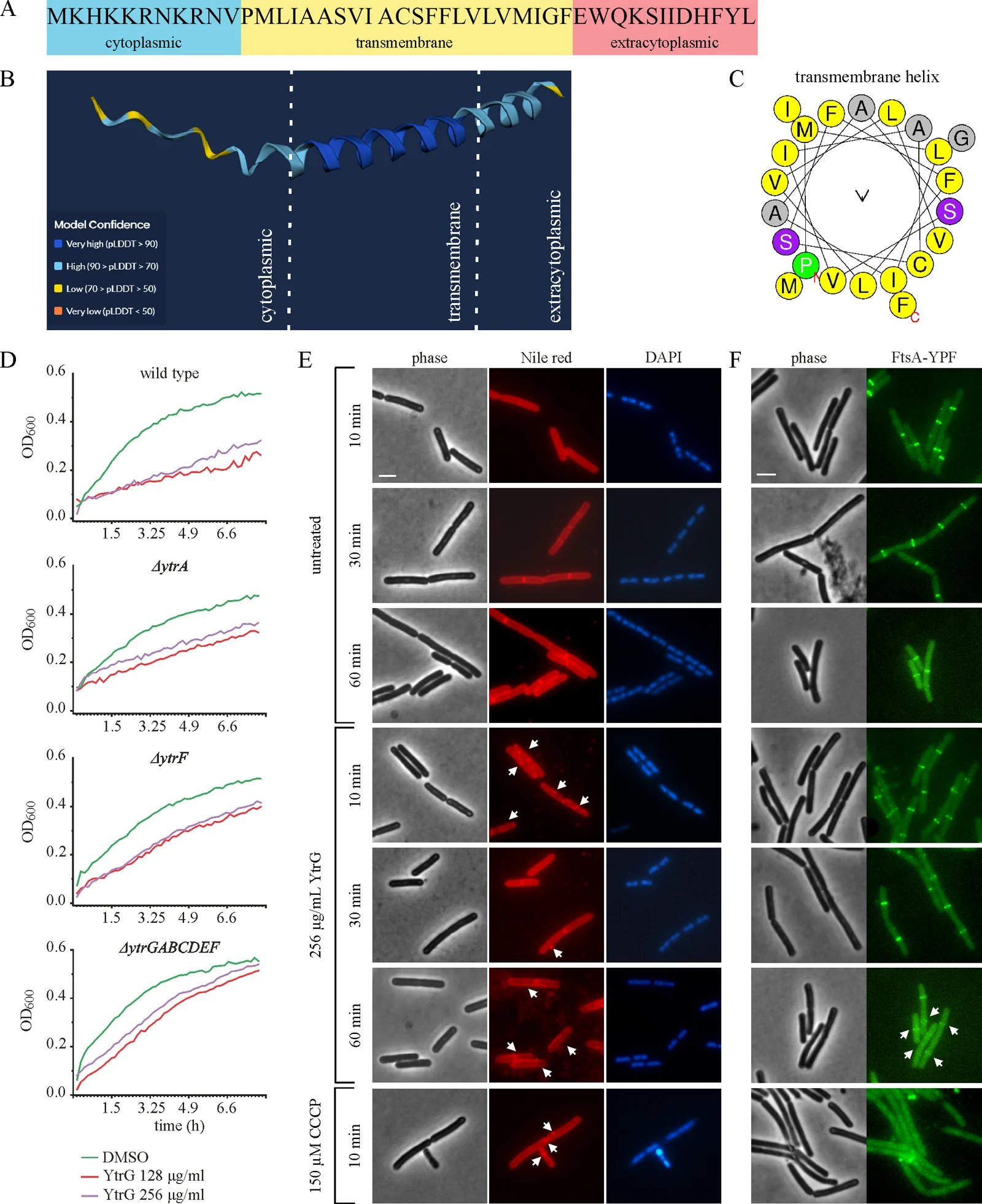
YtrG is a small transmembrane protein with mild antimicrobial effects. (**A**) YtrG amino acid sequence and domain prediction according to InterProScan topology (12). (**B**) AlphaFold2 (Neurosnap) structure prediction of YtrG (13–15). The full structural prediction data are available here: https://neurosnap.ai/job/662f9fcd89b5c0d5cabbf6e9?share=662fbdb689b5c0d5cabbf77d. (**C**) Helical wheel prediction of the transmembrane domain according to Heliquest (16). (**D**) Growth of *B. subtilis* 168CA (wild type), PH5 (∆*ytrA*), PD1 (∆*ytrF*), and GP2646 (∆*ytrGABCDEF*) in the presence of synthetic YtrG peptide. (**E**) Bacterial cytological profiling (BCP) of *B. subtilis* 168CA treated with 256 µg/mL synthetic YtrG peptide for 10 min. (**F**) Effects on the membrane potential-sensitive cell division protein FtsA. *B. subtilis* PG62 (*Pspac-yfp-ftsA*) was treated with 256 µg/mL synthetic YtrG peptide. Scale bar: 2 µM.

YtrG is a 45-amino acid-long protein (Fig. 1A) that is part of the *ytrGABCDEF* operon but not predicted to be part of the ABC transporter(s), neither in the YtrBCDEF nor the YtrB_2_CD and YtrE_2_F_2_ configurations (3, 11). Based on an InterProScan (12) analysis, the protein consists of a single transmembrane domain with an N-terminal cytosolic and a C-terminal extracellular domain (Fig. S1). AlphaFold2 (13–15) predicts YtrG to consist of a single α-helix with an unstructured N-terminus (Fig. 1B). These predictions are supported by HELIQUEST (16), confirming the predicted transmembrane domain to be highly hydrophobic (Fig. 1C; Fig. S2). This structure makes YtrG similar to type I toxins, small hydrophobic peptides of less than 60 amino acids containing a single transmembrane α-helix (9, 10). However, type I toxins are typically silenced by an antitoxin RNA, which is not predicted for the *ytrG* sequence. ToxinPred3.0 (17), a prediction tool for toxic peptides, recognized the first 10 amino acids of YtrG (MKHKKRNKRN) as a toxin motif. A BLAST search further identified a different 7-amino-acid motif (VLVMIGF) as overlapping with the type I toxin TxpA, a *B. subtilis* toxin that eliminates defective cells from biofilms (9, 10, 18, 19). However, comparing YtrG against the toxin-antitoxin database TATB3.0 (20) did not result in any hits. These mixed results suggest that, if *ytrG* should encode for a toxic gene product, it is not part of a known type of toxin-antitoxin system.

### YtrG has moderate antibacterial activity

To test whether YtrG has any direct-acting antibacterial effects, we used a synthetic full-length YtrG peptide carrying a C-terminal amidation to avoid stability issues in culture medium (MKHKKRNKRNVPMLIAASVIACSFFLVLVMIGFEWQKSIIDHFYL-NH_2_). We first performed minimal inhibitory concentration (MIC) assays at 37°C and 24°C. While we did not observe an MIC (=clear wells) up to 512 µg/mL, we did observe a concentration-dependent reduction in growth that already set in at much lower concentrations. Therefore, we ran acute shock assays with exponentially growing cells. Figure 1D shows the results for 37°C. Indeed, 128 µg/mL of YtrG peptide was sufficient to reduce the growth of wild-type *B. subtilis* by 50% compared to the dimethyl sulfoxide (DMSO) control. To test whether the *ytr* operon affects the toxicity of the YtrG peptide, we tested its activity on ∆*ytrA*, ∆*ytrF*, and ∆*ytrGABCDEF*. While all three strains were affected by the YtrG peptide, the growth-inhibitory effect was milder for ∆*ytrF* and ∆*ytrGABCDEF*, suggesting that the presence of a functional operon may aggravate the peptide’s effects. We also performed the same experiment at 24°C, where the peptide showed much higher activity. However, equal volumes of the solvent DMSO alone strongly impaired bacterial growth under these conditions so that no effects could be clearly attributed to YtrG (Fig. S3). We then wondered if YtrG would primarily inhibit growth of *B. subtilis* or of other, potentially competing bacteria. To assess this, we tested its activity against Gram-negative *E. coli* and Gram-positive *Macrococcus carouselicus*. While we could indeed observe activity against *E. coli* at 37°C, *M. carouselicus* was not affected. However, growth inhibition of *E. coli* (~25%) was considerably less than that of *B. subtilis* (~50%), suggesting that the YtrG peptide primarily affects its own source strain. At 24°C, we observed the same DMSO sensitivity that precluded an accurate assessment of the effects of YtrG on *B. subtilis* (Fig. S4 and S5).

We then asked ourselves whether constitutive expression of YtrG could be responsible for the small cell and lysis phenotypes observed with the different *ytr* mutants (7). This could make sense if the ABC transporter(s) would play a role in the membrane insertion or export of YtrG, since these mutations would lead to the constitutive expression of YtrG in cells with a dysfunctional transporter and, hence, its intracellular accumulation or faulty membrane insertion. To test this hypothesis, we performed bacterial cytological profiling (BCP), the method used previously to reveal small cells (7), of wild-type *B. subtilis* exposed to 256 µg/mL synthetic YtrG peptide (Fig. 1E). However, neither the small cell nor the lysis phenotypes were observed, and treated cells only displayed mild membrane aberrations. However, we noticed that the hydrophobic YtrG peptide interacted with membrane dyes, making it difficult to interpret this effect. Therefore, we employed green-fluorescent protein (GFP) fusions to rule out a possible effect on the membrane potential, which has been described for toxins and can trigger autolysis in *B. subtilis* (21). To this end, we assessed the localization of the cell division proteins FtsA and MinD, which require the membrane potential for their correct localization (22). However, the localization of MinD was undisturbed (Fig. S6), while effects on FtsA were mild and only visible after 60 min of treatment (Fig. 1F). These results show that YtrG does not collapse the membrane potential. In conclusion, while synthetic YtrG peptide possesses mild antibacterial properties, it does not appear to be a toxin that causes the observed small cell and lysis phenotypes.

### Phenotypic effects of YtrG

**Fig 2:**
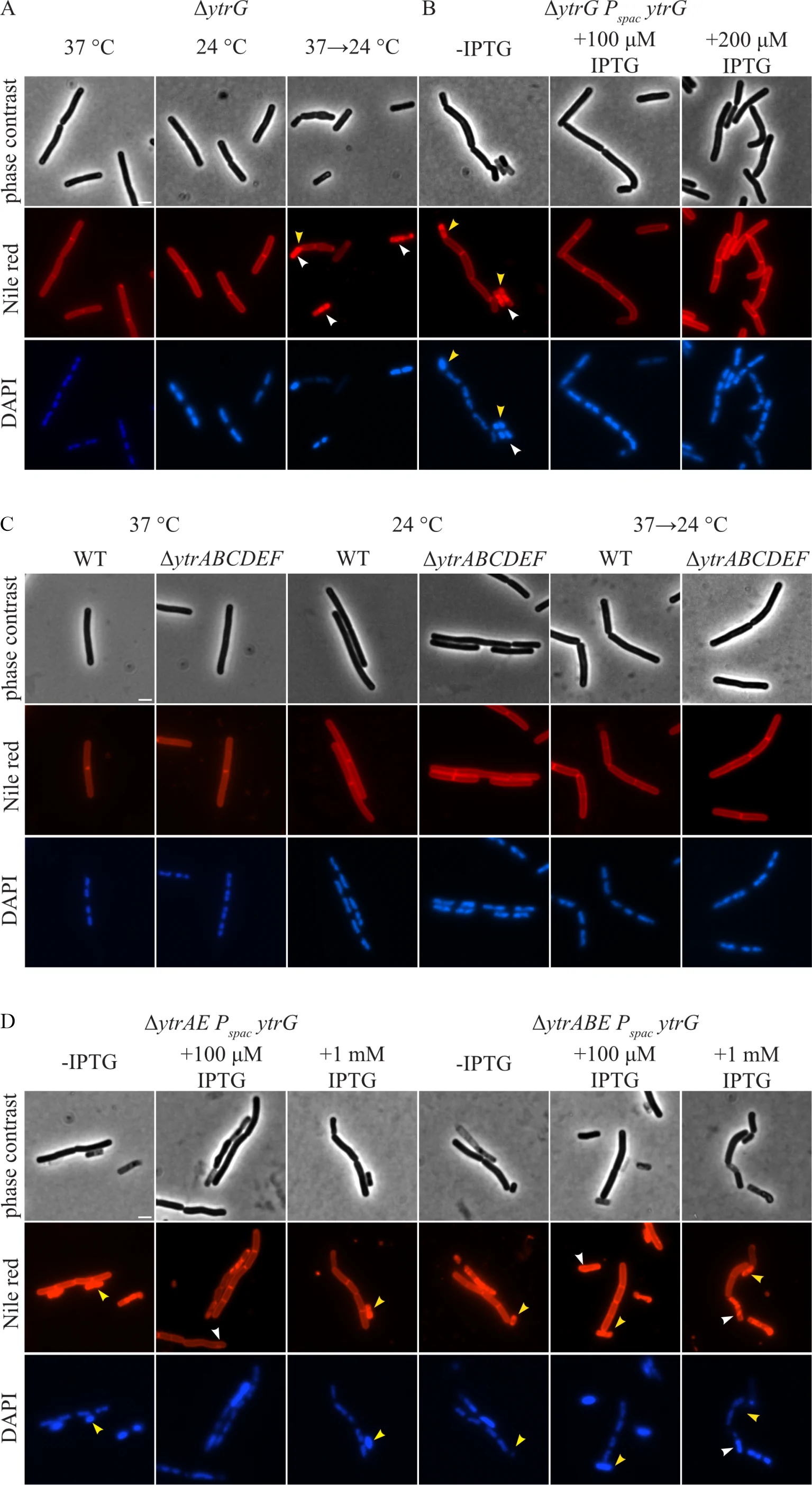
Phenotypic characterization of *B. subtilis* mutants. (**A and B**) IPTG-dependent compensation of the small cell and lysis phenotypes in a Δ*ytrG* mutant. (**A**) The small cell phenotype is visible in GP2699 (Δ*ytrG*) after a temperature shift from 37°C to 24°C. (**B**) IPTG-dependent compensation of the small cell phenotype in MS52 (Δ*ytrG Pspac-ytrG*). All images in panel B show 37°C→24°C shift conditions. Note that while small cells still occur, less cell lysis and concomitant membrane stress are observed in the presence of IPTG. (**C**) *B. subtilis* expressing *ytrG* (HB5447) as the only gene of the operon does not show small cells or any other aberrant phenotype compared to its corresponding WT strain (CU1065). (**D**) Ectopic expression of *ytrG* does not rescue small cell lysis phenotypes in Δ*ytrAB* (MS57) and Δ*ytrABE* (MS58). Scale bars: 2 µm. Yellow arrows indicate small cells. White arrows indicate membrane aberrations.

It is conceivable that YtrG needs to be present inside the cells to elicit these phenotypes, for example, to be correctly inserted into the cell membrane or to be proteolytically processed into an active peptide. Therefore, we constructed a strain expressing *ytrG* from an isopropyl β-D-1-thiogalactopyranosid (IPTG)-inducible promoter (MS48) and assessed whether the overproduction of YtrG is toxic in *B. subtilis*. However, we did not observe any IPTG-dependent growth defects when overexpression of *ytrG* was induced in the 168CA wild-type background (Fig. S7), suggesting that the toxin hypothesis is a dead end and that the observed antibacterial activity of externally applied YtrG peptide is coincidental. To confirm this, we constructed a Δ*ytrG* deletion strain (GP2699). If YtrG would be responsible for the small cell phenotype, it should disappear in the deletion strain. However, the small cell phenotype was still observed to an extent comparable to the *ytr* double mutants (7) (Fig. 2A).

**Fig 3:**
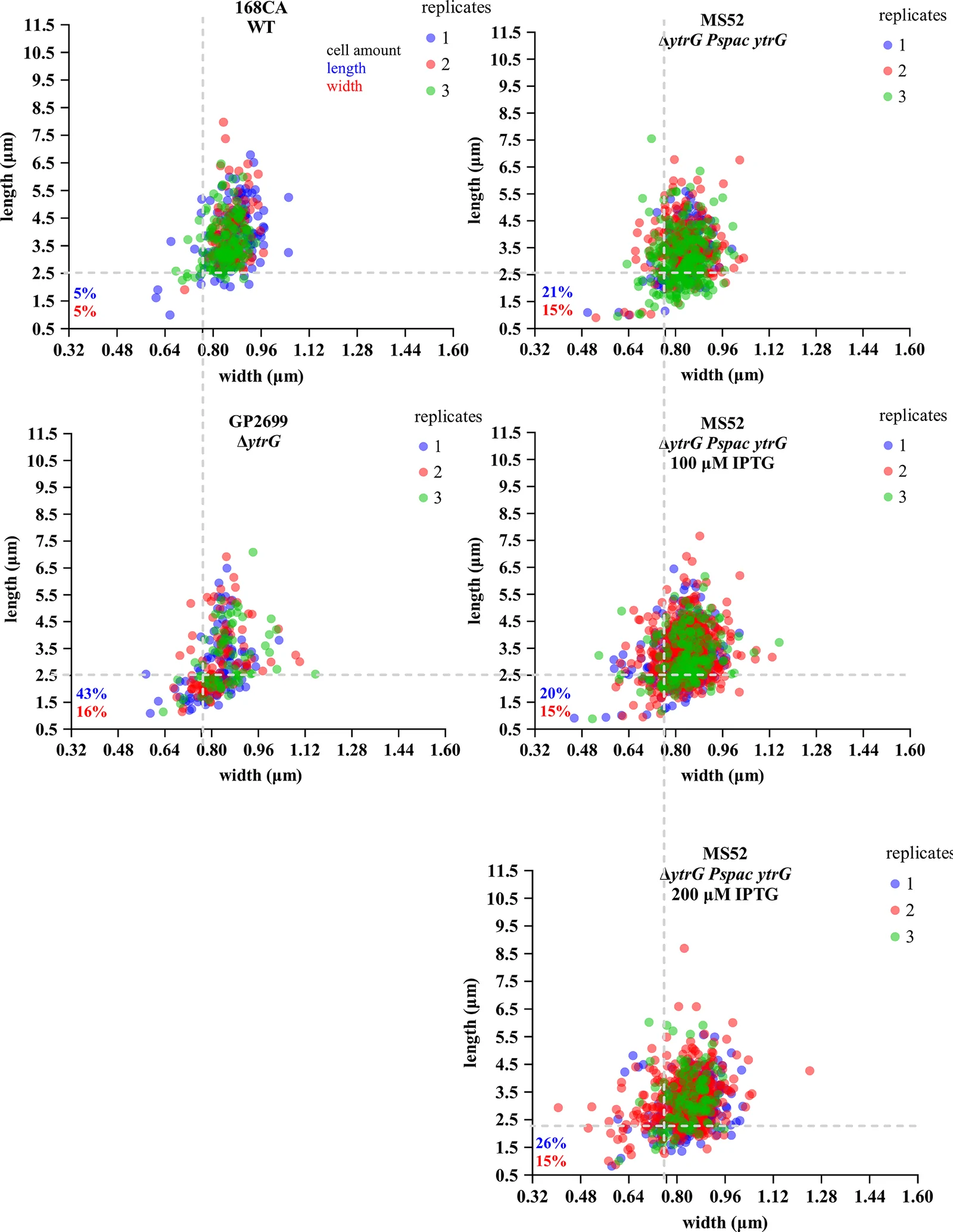
Quantification of cell length and width of *B. subtilis* 168CA (WT), GP2699 (Δ*ytrG*), and MS52 (Δ*ytrG Pspac-ytrG*). Blue, red, and green data points represent three biological replicates. Dotted lines represent cutoff values for the 95th quantile for the WT (95% of the WT cell population falling above the cutoff in length or width, respectively). Percentages show the proportion of cells outside of the 5% WT cutoffs for length (red) and width (blue).

When we expressed the *Pspac-ytrG* construct in the Δ*ytrG* deletion background (MS52), we observed an IPTG-dependent complementation of the lysis and membrane stress phenotypes (Fig. 2B). We also observed partial complementation of the small cell phenotype in terms of cell length (Fig. 3). However, MS52 already showed similar complementation without the addition of IPTG, suggesting that already very low expression of YtrG may cause this effect. We did not observe a visible phenotype of a strain expressing only YtrG (Δ*ytrABCDEF*, HB5447; Fig. 2C). Taken together, these observations suggest a scenario in which the expression of YtrG prevents the lysis and small cell phenotypes, the opposite of our initial hypothesis. However, the expression of *ytrG* in the Δ*ytrAB* (MS57) and Δ*ytrABE* (MS58) mutants did not reverse their small cell phenotypes (Fig. 2D), indicating that it is not simply YtrG levels alone that achieve this effect.

When we further characterized the phenotype of the Δ*ytrG* deletion strain, we noticed that the small cell phenotype was dependent on both growth phase and temperature shift. Thus, small cells could be observed under similar conditions as for the previously examined mutants that displayed this phenotype (7), with the strongest effects upon shift from 37°C to 24°C (Fig. 2). When we varied temperatures and dilutions, we observed that the small cell phenotype was not pronounced when cells were diluted from log-phase cultures, but very clear when diluted from stationary cultures, especially when combined with a temperature shift upon dilution (Fig. S8 and S9). This pattern extended to growth behavior. When the Δ*ytrG* mutant was diluted from stationary cultures, it displayed temperature-dependent growth defects similar to those previously reported for other *ytr* mutants (6) (Fig. S10). In contrast, when logarithmically growing cultures were diluted to the same starting optical density, no growth defects were observed (Fig. S11). These observations suggest that the small cell phenotype does not depend on cell density but rather on growth phase and is exacerbated by cold. These data are well-aligned with our previous interpretation that the *ytrGABCDEF* operon likely fulfills a function in adapting from slow to fast growth, possibly related to balancing cell wall synthesis and autolysis.

### Stress-induced membrane translocation of YtrB and YtrE

**Fig 4:**
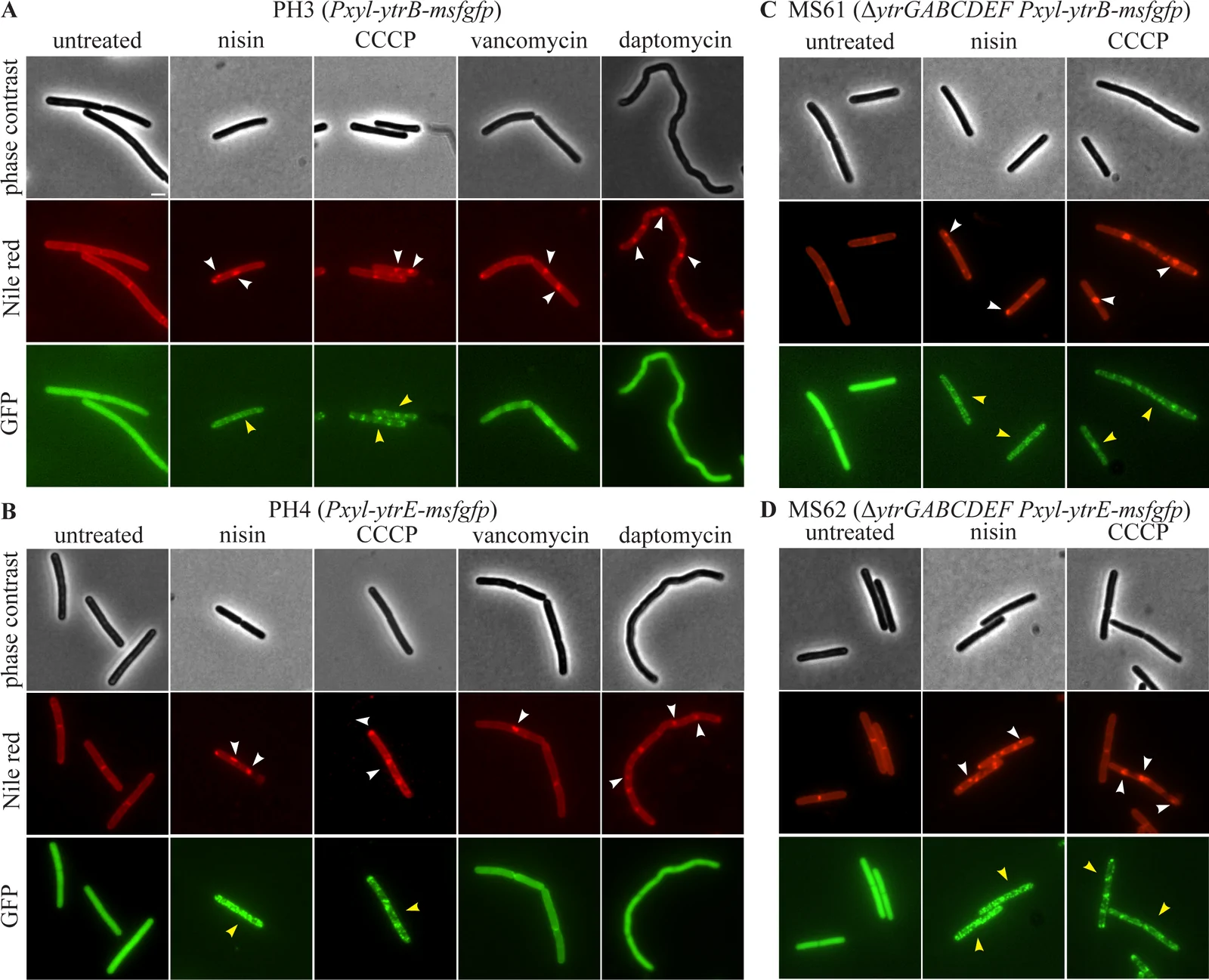
Fluorescence microscopy images of *B. subtilis* strains expressing GFP-tagged YtrB and YtrE. (**A**) *B. subtilis* WT expressing YtrB-msfGFP (PH3), (**B**) WT expressing YtrE-msfGFP (PH4), (**C**) Δ*ytrGABCDEF* expressing YtrB-msfGFP (MS61), and (**D**) Δ*ytrGABCDEF* expressing YtrB-msfGFP (MS62). Fusion protein expression was induced with 0.1% xylose. Scale bar: 2 µm. White arrows indicate membrane foci caused by antibiotics. Yellow arrows indicate cells with membrane-associated GFP foci.

While running structural analyses on possible Ytr transporter topologies, we observed that when we added YtrG to the YtrBCDEF configuration, AlphaFold3 predicted YtrE to interact with YtrD, resembling the YtrBCDEF topology suggested by Yoshida et al. (2, 7). However, in the absence of YtrG, it placed YtrE with YtrF, as in the newer structural predictions made by Mahendran et al. (3) and Yu et al. (4, 7). Indeed, the similar phenotypes of the Δ*ytrAB* and Δ*ytrAE* mutants (7), despite accumulating evidence for a YtrB_2_CD and YtrE_2_F_2_ topology, could point toward promiscuity of YtrB and YtrE toward YtrD and YtrF. We previously reported that a GFP fusion to YtrE did not localize to the cell membrane (6) and initially assumed that the fusion was simply not functional. The same was observed for YtrB (Fig. 4A and B). However, when cells expressing these fusions were stressed with membrane-active antibiotics, they translocated to the cell membrane and formed distinct membrane-associated foci (Fig. 4A and B). This was not the case when free cytosolic GFP was expressed (Fig. S12A), indicating that this phenotype is not due to non-specific protein aggregation. Together, these observations prompted the hypothesis that the transporter(s) encoded by the *ytrGABCDEF* operon could possibly undergo stress-induced structural changes. In this scenario, YtrG could act as a molecular switch inducing a structural change that affects subunit interactions. To assess this possibility, we transformed the GFP fusion constructs into the Δ*ytrGABCDEF* deletion background and tested whether their localization still changed under stress conditions (Fig. 4C and D). However, this was still the case. Additionally, we assessed the localization of YtrD under the same conditions and observed localization patterns typical for the antibiotic treatment conditions (6) but distinct from the patterns observed for YtrB and YtrE (Fig. S12B). These findings suggest that the localization change of YtrB and YtrE does not depend on the presence of any *ytr* gene and is likely related to a physical change in the membrane and/or cytosolic environment that stimulates protein accumulation and membrane translocation. Whether this observation is of any relevance to the function of the Ytr transporter(s) remains to be addressed in future studies.

### Conclusion

Here, we assessed the role of YtrG in the previously observed small cell and lysis phenotypes induced by the overexpression of the *ytrGABCDEF* operon, pursuing two separate hypotheses, namely a role as a toxin or as a modulator of Ytr transporter function. While we could establish that the YtrG peptide does indeed possess mild antimicrobial properties, we could conclude that it is not a new type of toxin and does not cause the small cell phenotype. On the contrary, its absence exacerbated small cell lysis, suggesting that it may play a role in counteracting rather than promoting cell lysis. It should be noted that Δ*ytrG* was the only *ytr* single mutant that showed such strong small cell and lysis phenotypes, on par with mutants overexpressing an incomplete operon (7), suggesting that its gene product plays a critical role in the function of the operon. We could not confirm the hypothesis that YtrG modulates Ytr channel interactions, a notion prompted by changes in YtrB and YtrE localization under conditions that induce the operon. However, this localization change was independent of the presence of the *ytr* operon and therefore seems to be unrelated. However, this observation does not rule out that YtrG may still play a role in modulating operon function in another capacity. Further studies are needed to elucidate the role of YtrG in the function of the *ytrGABCDEF* operon.

## MATERIALS AND METHODS

### Bioinformatics

All *B. subtilis* gene sequences were obtained from the SubtiWiki database (11). The YtrG domain, homology, and structural predictions were based on InterProScan (12), BlastP (23–26), AlphaFold2 (Neurosnap) (13–15), and Heliquest (16). Toxin prediction analysis was based on the ToxinPred3.0 (17) tool and the TATB3.0 (20) database.

### Strains and growth conditions

Strains and plasmids used in this study are listed in Table S1. Unless otherwise stated, all cultures were grown in lysogeny broth (LB) under constant agitation. Experiments were performed at constant 37°C or 24°C, or after a temperature shift from 37°C to 24°C.

### Strain and plasmid construction

Plasmid pPH1 (pMW1-*Pxyl-ytrB-msfgfp*) was constructed by Gibson assembly (27) (GeneArt Gibson Assembly HiFi Kit, Invitrogen) as described previously (6). Plasmid pMS31 was constructed by removing the 78 bp multiple cloning site in plasmid pMW1 (28) through inverted PCR. pTWIST3_*Pspac-ytrG* was constructed by Twist Bioscience (https://www.twistbioscience.com/) through synthesis of a 2,363 bp gene fragment containing *Pspac-ytrG* and *lacI* followed by insertion into plasmid pMW1 (28). Deletion of *ytrG* (strain GP2699) was achieved by transformation of *B. subtilis* 168CA with PCR products containing a kanamycin resistance cassette flanked by up- and downstream fragments of the target gene as described previously (29–31). All plasmids were verified by sequencing (Eurofins MWG). Primer sequences for all constructs are listed in Table S2. *B. subtilis* strains were transformed using a standard starvation protocol (32). All transformed strains were confirmed by PCR.

### Minimal inhibitory concentration

MICs of synthetic YtrG peptide against *B. subtilis* 168CA were determined in standard microdilution assays according to guidelines from the Clinical and Laboratory Standards Institute (33) with minor modifications. In short, serial twofold dilutions of YtrG peptide in LB were inoculated with 5 × 10^5^ colony-forming units (CFU) per milliliter of exponentially growing *B. subtilis* 168CA cultures and incubated at 24°C or 37°C for 16 h. MICs were determined as the lowest concentration inhibiting visible growth.

### Growth experiments

All growth curves were recorded on a temperature-controlled BMG Clariostar Plus plate reader under constant shaking. Optical density readings were taken in 5-min intervals. For YtrG peptide acute shock experiments, overnight cultures of *B. subtilis* 168CA were diluted to an OD_600_ of 0.05 and grown until the early exponential growth phase (OD_600_ 0.3–0.8). Cultures were then adjusted to an OD_600_ of 0.3 and transferred to sterile 96-well plates containing different amounts of YtrG peptide. To assess inhibitory effects of YtrG peptide on *E. coli* W3110 and *M. carouselicus*, LB medium containing different concentrations of YtrG peptide was inoculated with 5 × 10^5^ CFU/mL. DMSO (solvent control) was included in all experiments. Growth curves were recorded at constant 24°C and 37°C. To assess the effects of YtrG overexpression, LB containing different concentrations of IPTG was inoculated with 5 × 10^5^ CFU/mL of *B. subtilis* MS48 (*amyE::spc Pspac-ytrG lacI*) or 168CA. Growth was recorded at constant (24°C and 37°C). To assess the growth behavior of GP2699 (Δ*ytrG*) under different conditions, overnight cultures were diluted to an OD_600_ of 0.05 and either directly transferred to 96-well plates for growth experiments, or first allowed to grow to the exponential growth phase (OD_600_ 0.3–0.8) before re-adjustment to an OD_600_ of 0.05. Growth was then monitored at the same temperature (24°C and 37°C) or after a temperature shift from 37°C to 24°C.

### Microscopy

All microscopy samples (0.5 µL) were spotted on glass slides coated with a thin film of 1.2% agarose and covered with a poly-L-dopamine-coated coverslip as described previously (34). Samples were imaged on a Nikon Eclipse Ti2 inverted fluorescence microscope equipped with a CFI Plan Apochromat objective (DM Lambda 100× Oil N.A. 1.45, W.D. 0.13 mm, Ph3), a Lumencor Sola SE II FISH 365 light source, a Photometrics PRIME BSI camera, an Okolab incubator, and Nis ELEMENTS AR 5.21.03 software. All microscopy images were processed and analyzed with Fiji (35).

### Bacterial cytological profiling

BCP was performed as described in Schäfer et al. (36). In short, *B. subtilis* strains were grown to an OD_600_ of 0.3 and subsequently stained with 0.5 μg/mL Nile red (Invitrogen, membrane stain) and 1 μg/mL 4′,6-diamidino-2-phenylindole (DAPI) (Invitrogen, DNA stain) for 5 min. To examine the effects of synthetic YtrG peptide, *B. subtilis* 168CA was grown at 37°C until an OD_600_ of 0.3 and subsequently treated with 256 µg/mL YtrG for 5, 25, and 55 min prior to staining and microscopy. Small cells were quantified based on cell length and width using Fiji and the ImageJ plugin MicrobeJ (35, 37). The MicrobeJ parameters for bacterial detection in phase contrast were set to default segmentation with an area of 0.4 μm^2^-max. All other parameters remained at default settings. All chained cells were assessed and separated using the MicrobeJ manual editing interface (37). The 95th WT quantiles of cell length and width were determined by sorting the respective data tables. All cells with values outside of the 95th WT quantiles, either in length or width, were categorized as small cells. Distribution plots were generated and processed in GraphPad Prism version 10.6.1.

### Protein localization microscopy

To examine the effects of synthetic YtrG peptide on protein localization, *B. subtilis* PG62 (*Pspac-yfp-ftsA*) and TB35 (*Pxyl-gfp-minD*) were grown at 37°C until an OD_600_ of 0.3 and subsequently treated with 256 µg/mL YtrG for 10, 30, and 60 min. For localization of YtrB-GFP and YtrE-GFP, strains PH3 (*Pxyl-ytrB-msfgfp*) and PH4 (*Pxyl-ytrE-msfgfp*) were grown at 37°C until an OD_600_ of 0.3 and subsequently treated with 2 µg/mL daptomycin for 60 min, 100 µM carbonyl cyanide m-chlorophenylhydrazone (CCCP) for 10 min, 0.75 µg/mL nisin for 10 min, or 0.5 µg/mL vancomycin for 10 min. MS39 (*Pxyl-msfgfp*), MS42 (*Pxyl-ytrD-msfgfp*), MS61 (Δ*ytrGABCDEF Pxyl-ytrB-msfgfp*), and MS62 (Δ*ytrGABCDEF Pxyl-ytrE-msfgfp*) were grown analogously but only treated with 100 µM CCCP or 0.75 µg/mL nisin for 10 min. Cultures for daptomycin treatment were grown in the presence of 1.25 mM CaCl_2_. Untreated controls were included for all conditions and time points. All GFP-expressing strains were grown in the presence of appropriate inducer concentrations (0.1 mM IPTG for PG62, 0.25% xylose for MS42, and 0.1% xylose for TB35, PH3, PH4, MS39, MS61, and MS62).

### Phase contrast microscopy

For assessment of small cells at different ODs, strains were either grown at constant 24°C or 37°C, or shifted from 37°C to 24°C upon dilution of overnight cultures. Samples were taken at an OD_600_ of 0.1, 0.4, and 2, spotted on agarose-covered glass slides, and examined by phase contrast microscopy. To examine the effects of temperature shifts on cells adjusting from stationary to exponential growth, overnight cultures of GP2699 (Δ*ytrG*) grown at 37°C were diluted to an OD_600_ of 0.05 and split into two groups. The first group was allowed to grow at 37°C until reaching an OD_600_ of 0.1, 0.4, and 2, respectively, and was subsequently shifted to 24°C. Images were taken 1, 2, 3, and 4 h after the shift, as well as after overnight incubation. The second group was shifted immediately after dilution, and images were taken after cultures had reached an OD_600_ of 0.1 and 0.4, respectively, as well as after overnight incubation.

## Data Availability

All data, metadata, and methods used to reach the conclusions of this study are included in the main text and Supplemental Information. Raw image files will be made available upon request.

## SUPPLEMENTAL MATERIAL

Supplemental tables and figures spectrum.02912-26-s0001.pdf Tables S1 and S2, and Fig. S1 to S12.

ASM does not own the copyrights to Supplemental Material that may be linked to, or accessed through, an article. The authors have granted ASM a non-exclusive, world-wide license to publish the Supplemental Material files. Please contact the corresponding author directly for reuse.
