# Supplementary material for: YtrG is a functional part of the ytrGABCDEF operon and contributes to phenotypic heterogeneity in Bacillus subtilis
Source: Microbiol Spectr. 2026 Oct 6;14(11):e02912-26. doi: 10.1128/spectrum.02912-26 (PMC13729164; doi:10.1128/spectrum.02912-26)
Supplement: Supplementary file 1 [file spectrum.02912-26-s0001.pdf]

Supplementary Information

YtrG is a functional part of the *ytrGABCDEF* operon and contributes to phenotypic heterogeneity in *Bacillus subtilis*

Margareth Sidarta[1,2], Pauline Hammer úr Skúoy[1], Martin Benda[3,4], Jörg Stülke[3], Michaela Wenzel[1,2]*

[1]Division of Molecular Bioscience, Department of Life Sciences, Chalmers University of Technology, Gothenburg, Sweden

[2]Centre for Antibiotic Resistance Research in Gothenburg (CARe), Gothenburg, Sweden

[3]Department of General Microbiology, Institute of Microbiology and Genetics, Georg-August University Göttingen, Göttingen, Germany

[4]Department of Parasitology, Faculty of Science, BIOCEV, Charles University, Prague, Czech Republic

*Corresponding author: Michaela Wenzel (wenzelm@chalmers.se)

Supplementary tables

Table S1: Strains and plasmids used in this work.
Table S2: Primers used in this work.

Supplementary figures

Figure S1: Results of the InterProScan analysis of the YtrG protein sequence.
Figure S2: Full HELIQUEST results of the YtrG transmembrane domain.
Figure S3: Growth curves of *B. subtilis* wild type and *ΔytrA*, *ΔytrF*, and *ΔytrGABCDEF* deletion mutants after acute shock with synthetic YtrG peptide
Figure S4: Growth curves of *E. coli* and *M. carouselicus* after treatment with synthetic YtrG peptide.
Figure S5: DMSO controls corresponding to growth curves of *E. coli* and *M. carouselicus* after shown in **Figure S4**.
Figure S6: Effects of synthetic YtrG peptide on the membrane potential-sensitive cell division protein MinD.
Figure S7: Growth of MS48 (*Pspac-ytrG*) and 168CA (WT) in presence of different IPTG concentrations.
Figure S8: Phase contrast microscopy of *B. subtilis* 168CA (WT) subjected to a temperature shift from 37 to 24 °C at different ODs.
Figure S9: Phase contrast microscopy of *B. subtilis* GP2699 (Δ*ytrG*) subjected to a temperature shift from 37 to 24 °C at different ODs.
Figure S10: Growth curves of *B. subtilis* 168CA (WT) and *ytr* mutant strains under different temperature conditions.
Figure S11: Growth curves of *B. subtilis* 168CA (WT) and GP2966 (Δ*ytrG*) under different temperature conditions.
Figure S12: Fluorescence microscopy images of *B. subtilis* MS39 (*Pxyl-msfgfp*) and MS42 (*Pxyl-ytrD-msfgfp*) treated with 0.5 µg/mL nisin and 100 µM CCCP for 10 min.

Supplementary references

Table S1: Strains and plasmids used in this work. gfp: green-fluorescent protein, msfgfp: monomeric superfolder gfp, MCS: multiple cloning site

strain	genotype	origin
B. subtilis 168CA	*trpC2*	[1]
B. subtilis BLMS3	*trpC2 ΔytrA::ery ΔytrCD*	[2]
B. subtilis CU1065	*trpC2 attSPβ*	[3]
B. subtilis GP2646	*trpC2 ΔytrGABCDEF::ery*	[2]
B. subtilis GP2699	*trpC2 ΔytrG::aphA3 (*without terminator)	this work
B. subtilis GP3193	*trpC2 ΔytrA::ery ΔytrB*	[2]
B. subtilis GP3196	*trpC2 ΔytrA::ery ΔytrE*	[2]
B. subtilis GP3206	*trpC2 ΔytrA::ery ΔytrB ΔytrE*	[2]
B. subtilis HB5447	*trpC2 attSPβ ytrABCDEF::kan*	[3]
B. subtilis JH642	*trpC2 pheA1*	[4–7]
B. subtilis MS39	*trpC2 pheA2 amyE::spc Pxyl-msfgfp*	pMS31→JH642
B. subtilis MS42	*trpC2 amyE::spc Pxyl-ytrD-msfgfp*	[8]
B. subtilis MS48	*trpC2 amyE::spc Pspac-ytrG lacI*	pTWIST3_*Pspac-ytrG*→168CA
B. subtilis MS52	*trpC2 ΔytrG::aphA3 amyE::spc Pspac-ytrG lacI*	GP2699→MS48
B. subtilis MS57	*trpC2 ΔytrA::ery ΔytrB amyE::spc Pspac-ytrG lacI*	GP3193→MS48
B. subtilis MS58	*trpC2 ΔytrA::ery ΔytrB ΔytrE amyE::spc Pspac-ytrG lacI*	GP3206→MS48
B. subtilis MS61	*trpC2 ΔytrGABCDEF::ery amyE::spc Pxyl-ytrB-msfgfp*	GP2646→PH3
B. subtilis MS62	*trpC2 ΔytrGABCDEF::ery amyE::spc Pxyl-ytrE-msfgfp*	GP2646→PH4
B. subtilis PD1	*trpC2 ΔytrF::kan*	[8]
B. subtilis PD2	*trpC2 ΔytrD::kan*	[8]
B. subtilis PD3	*trpC2 ΔytrC::kan*	[8]
B. subtilis PG62	*trpC2 aprE::Pspac-yfp-ftsA spc*	[9]
B. subtilis PH1	*trpC2 ΔytrB::kan*	[8]
B. subtilis PH2	*trpC2 ΔytrE::kan*	[8]
B. subtilis PH3	*trpC2 amyE:.spc Pxyl-ytrB-msfgfp*	pPH1→168CA
B. subtilis PH4	*trpC2 amyE::spc Pxyl-ytrE-msfgfp*	[8]
B. subtilis PH5	*trpC2 ΔytrA::ery*	[8]
B. subtilis TB35	*trpC2 amyE::spc Pxyl-gfp-minD*	[10]
E. coli TOP10	*F–mcrA Δ(mrr-hsdRMS-mcrBC) φ80lacZΔM15 ΔlacX74 recA1 araD139 Δ(ara-leu)7697 galU galK λ–rpsL(StrR) endA1 nupG*	ThermoFisher
E. coli W3110	*F- λ- thyA36 deoC2 IN1*	[11]
M. carouselicus		[12]
**plasmids**		
pMS31	*bla amyE::spc Pxyl-msfgfp* (without MCS)	this work
pMW1	*bla amyE::spc Pxyl-msfgfp*	[13]
pPH1	*bla amyE::spc Pxyl-ytrB-msfgfp*	this work
pTWIST3_*Pspac-ytrG*	*bla amyE::spc Pspac-ytrG*	this work

Table S2: Primers used in this work.

DNA template	primer	primer sequence	PCR product
Gibson assembly: vector backbone linearization			
pMW1	MWP1	ATGAGCAAAGGAGAAGAACTTTTC	linear pMW1
	Abs1	CCTAGGAATCTCCTTTCTAGATGC	
Gibson assembly: insert amplification			
168CA	PHP16	CTAGAAAGGAGATTCCTAGGATGATTGAACTGCGGCAGC	*ytrB* insert
	PHP17	AGTTCTTCTCCTTTGCTCATGCCGGAGCCGGAGCCGGAG CCGGAGCCGGAGCCGGACCATCTCCGCTTCCCTCC	
Inverted PCR: pMW1 MCS region removal			
pMW1	MSP144	P-ATGAGCAAAGGAGAAGAACTTTTC	linear pMS31
	MSP145	P-CCTAGGAATCTCCTTTCTAGATGC	
Plasmid and strain construction and confirmation			
pTWIST3_ Pspac-ytrG	MSP63	ATTAGTGACATTTGCATGCTTCAAAG	seq primer
pMS31	MSP63	ATTAGTGACATTTGCATGCTTCAAAG	seq primer
	TerS22	GGCAGACATGGCCTGCCCGGTT	seq primer
	TerS21	GGGCAACAAACTAATGTGCAA	152 bp
	Abs5	GAAAATTTGTGCCCATTAACATCACCATC	
pPH1	TerS21	GGGCAACAAACTAATGTGCAA	1225 bp
	PHP18	CATGCCGTTTCATGTGATCC	
GP2699	MB118	CCTATCACCTCAAATGGTTCGCTGCACTTAATACAATAAAT ACTTTGACTCACA	upstream forward
	MB171	CCTATCACCTCAAATGGTTCGCTGAACTGCCTCCCTTAATG TCAATAT	upstream reverse
	MB172	CCGAGCGCCTACGAGGAATTTGTATCGAAGGTTCAGATCG TATAGAAAGTAGG	downstream forward
	MB173	GCCTTGTCACCGCATCAATAC	downstream reverse
MS39, MS61, MS62, PH3	Absp12	CTGATTTATGAACAAAAAAGAAACCATCATTG	2567 bp (MS39); 3482 bp (MS61, PH3); 3299 bp (MS62)
	Abs5	GAAAATTTGTGCCCATTAACATCACCATC	
MS48, MS52, MS57, MS58	Absp11	GTGATAATTTTAAATGTAAGCGTTAACAAAATTC	4000 bp
	Absp12	CTGATTTATGAACAAAAAAGAAACCATCATTG	
MS52	MSP164	GTAGGTGAAGCAGATGGATTTTAATG	2845 bp
	MSP166	GGATTAAGCGAAGCATCGTCG	
MS57, MS58	MSP101	GCTCATTGGCATTACTTTTAATGGC	3746 bp (MS57); 3248 bp (MS58)
	PHP20	CGAAGTCGTATCCTACTACGATTTC	
MS61, MS62	MSP101	GCTCATTGGCATTACTTTTAATGGC	964 bp
	MSP161	TTCCACAAGCTTCTGATCAGC	

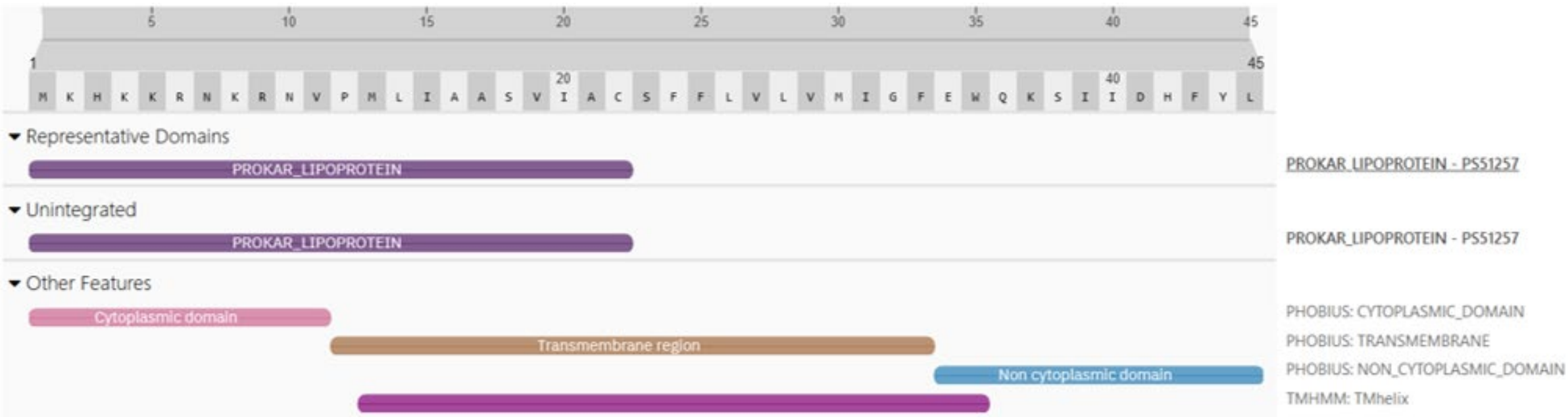

Figure S1: Results of the InterProScan[14] analysis of the YtrG protein sequence.

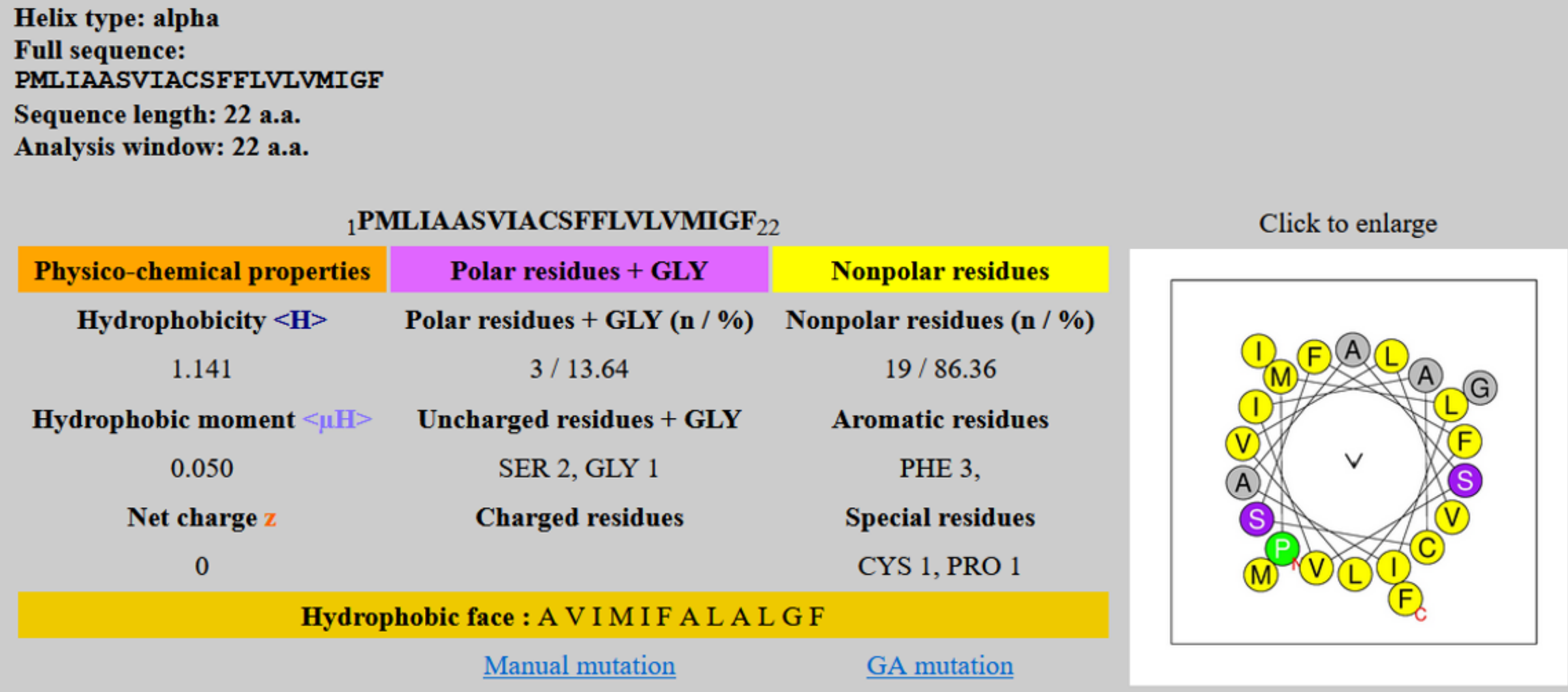

Figure S2: Full HELIQUEST[15] results of the YtrG transmembrane domain sequence.

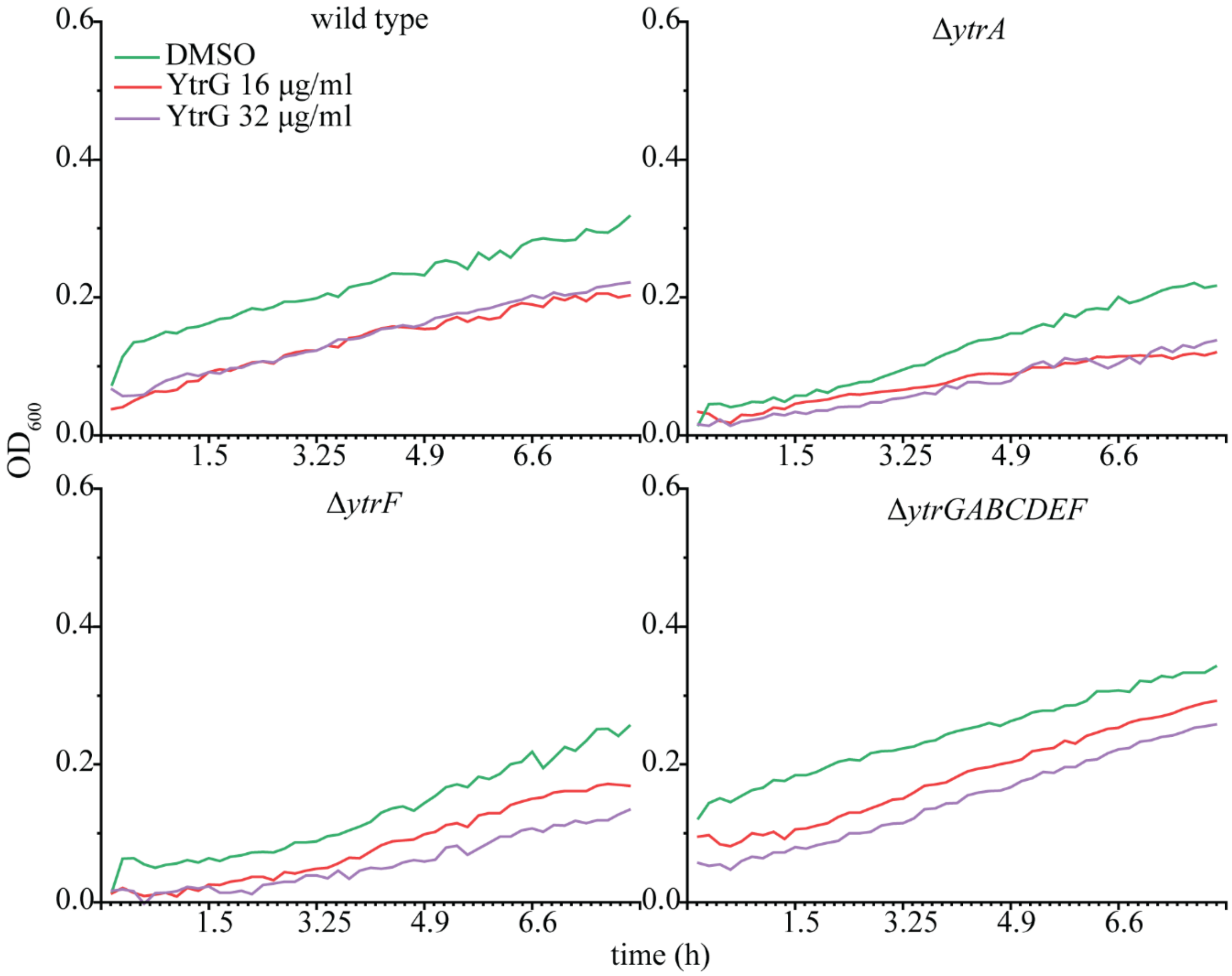

Figure S3: Growth curves of *B. subtilis* wild type and *ΔytrA* (PH5), *ΔytrF* (PD1), and *ΔytrGABCDEF* (GP2646) deletion mutants after acute shock with synthetic YtrG peptide. Cells were grown at 24 °C.

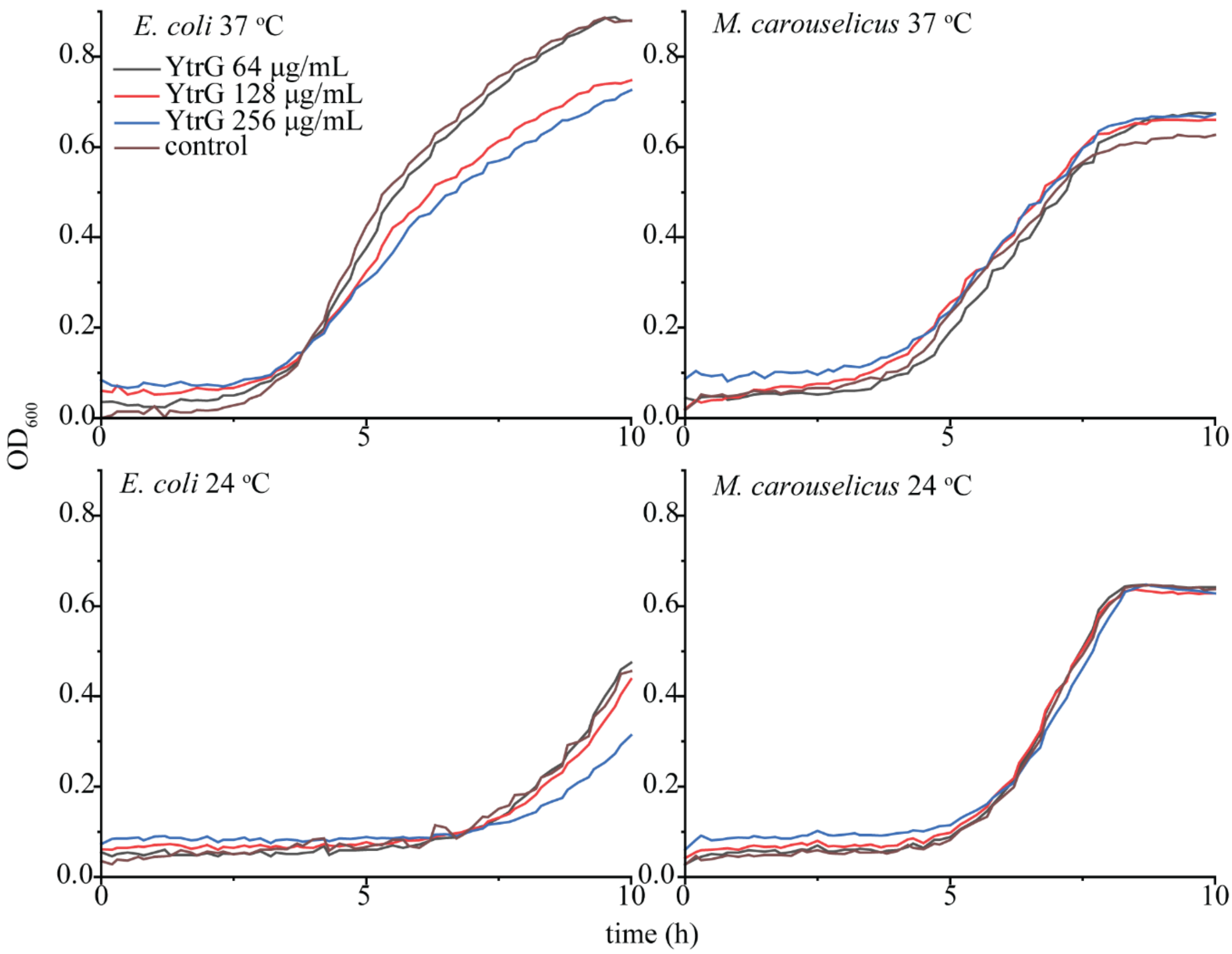

Figure S4: Growth curves of *E. coli* W3110 and *M. carouselicus* after treatment with synthetic YtrG peptide. Cells were grown in LB at 37 °C (top row) and 24 °C (bottom row).

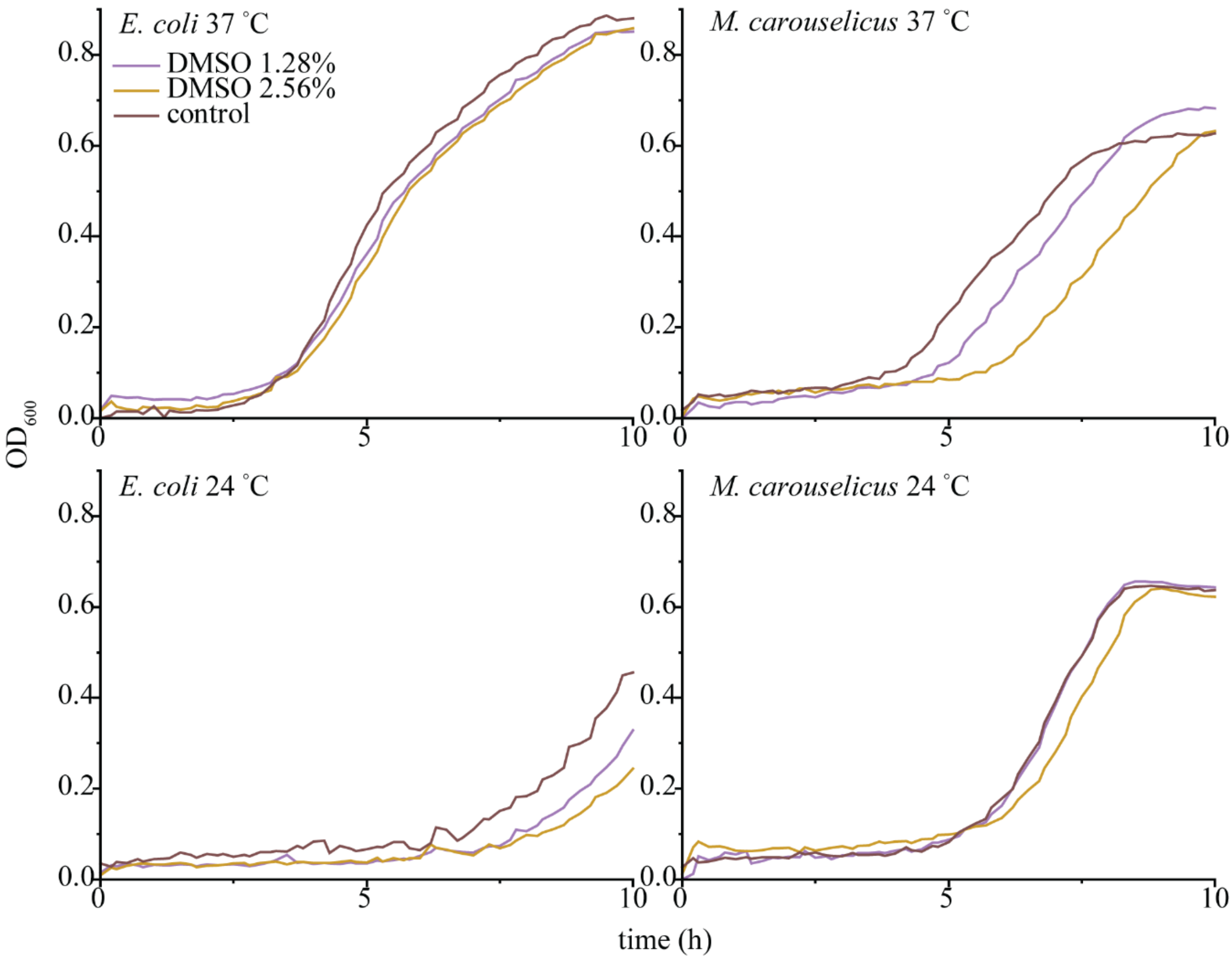

Figure S5: DMSO controls corresponding to growth curves of *E. coli* W3110 and *M. carouselicus* shown in **Figure S4**. Cells were grown in LB at 37 °C (top row) and 24 °C (bottom row).

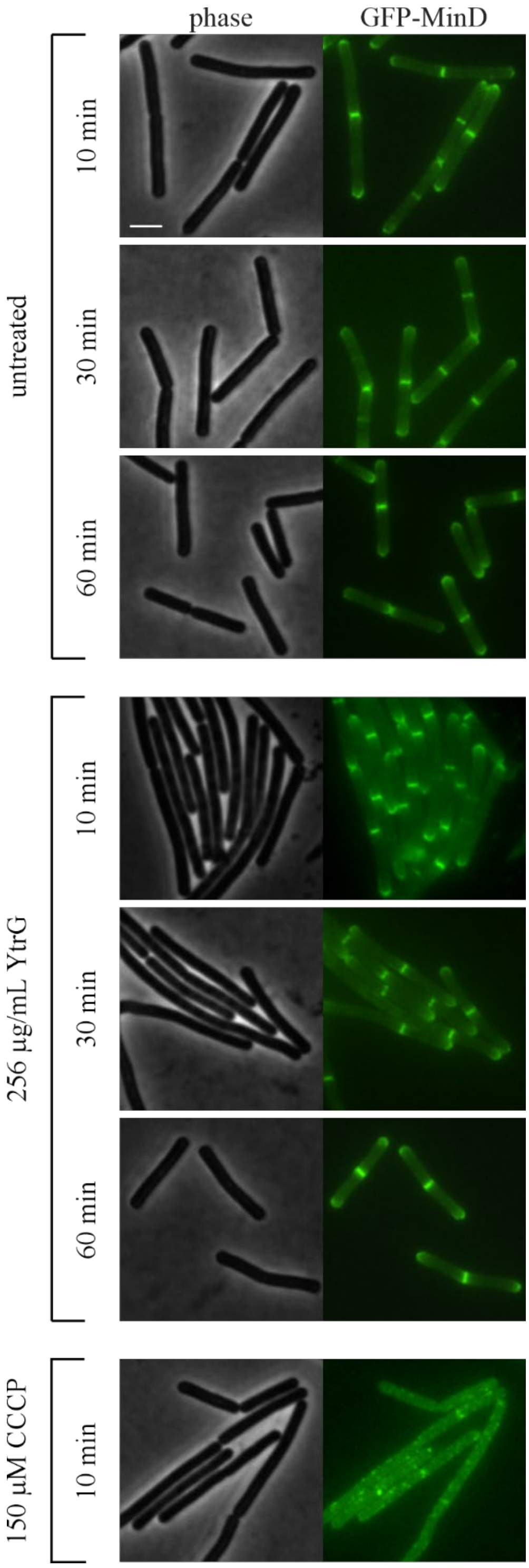

Figure S6: Effects of synthetic YtrG peptide on the membrane potential-sensitive cell division protein MinD. *B. subtilis* TB35 (*Pxyl-gfp-minD*) was treated with 256 µg/mL synthetic YtrG peptide. Scale bar 2 µm.

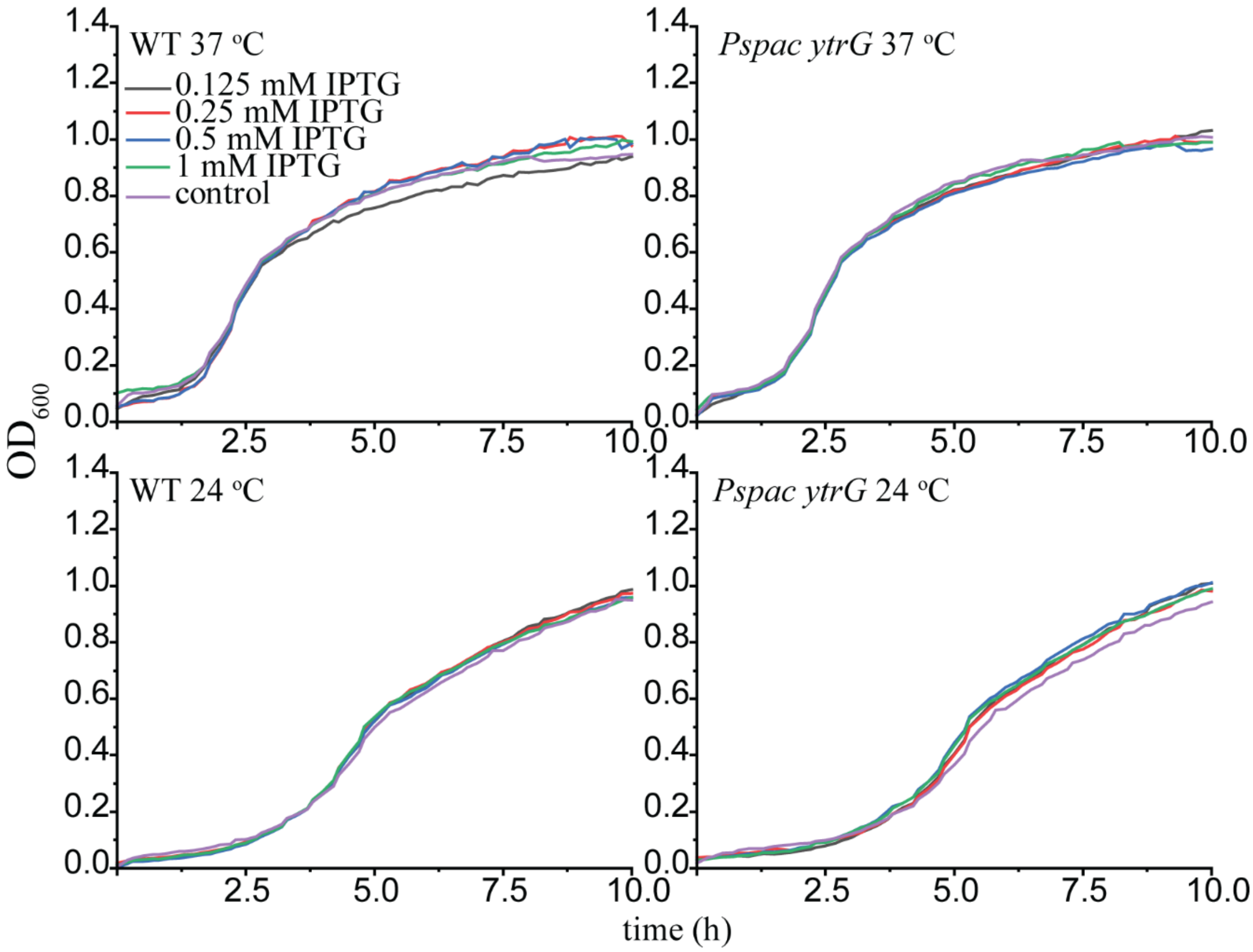

Figure S7: Growth of MS48 (*Pspac-ytrG*) and 168CA (WT) in presence of different IPTG concentrations.

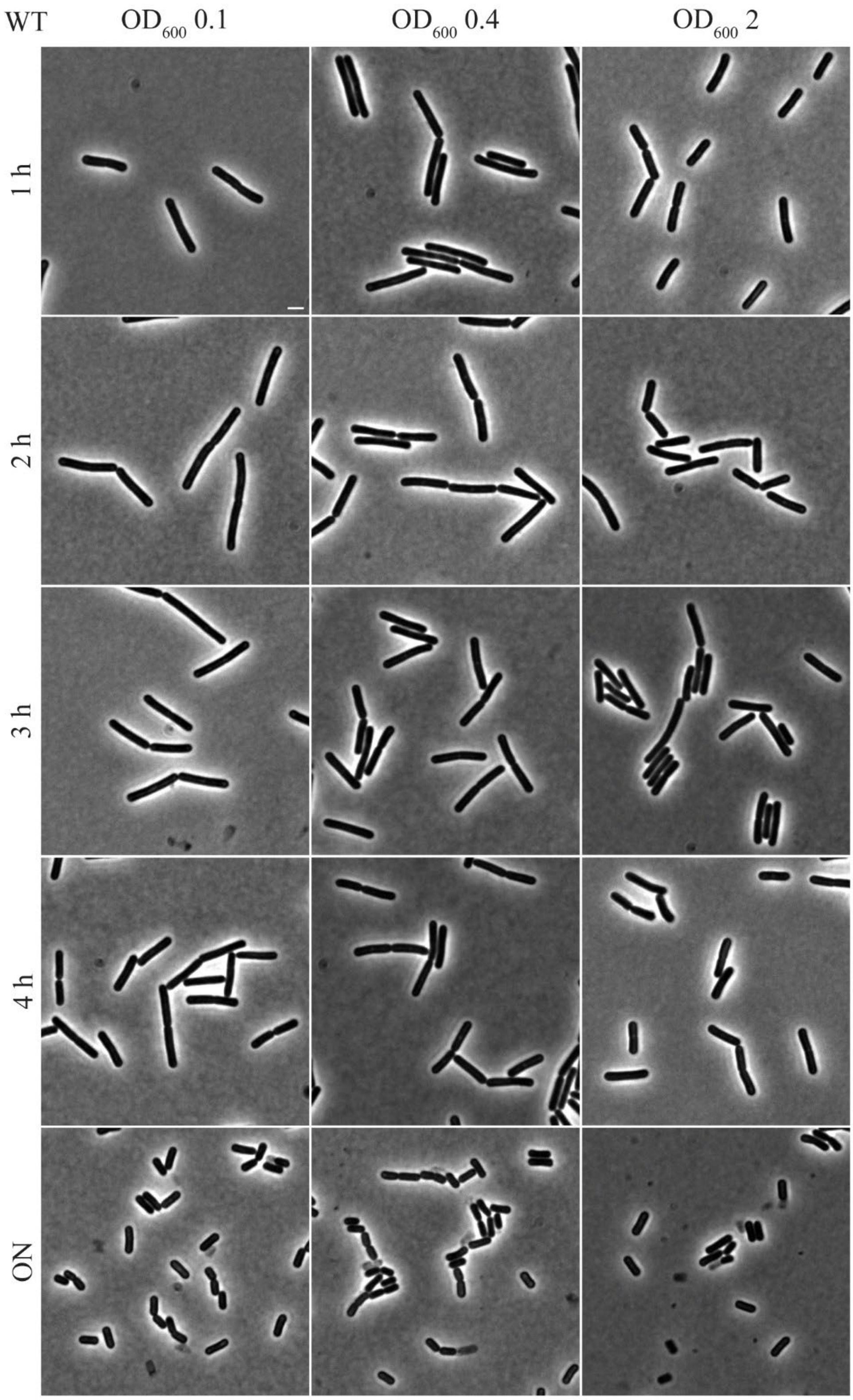

Figure S8: Phase contrast microscopy of *B. subtilis* 168CA (WT) subjected to a temperature shift from 37 to 24 °C at different ODs. Scale bar 2 μm.

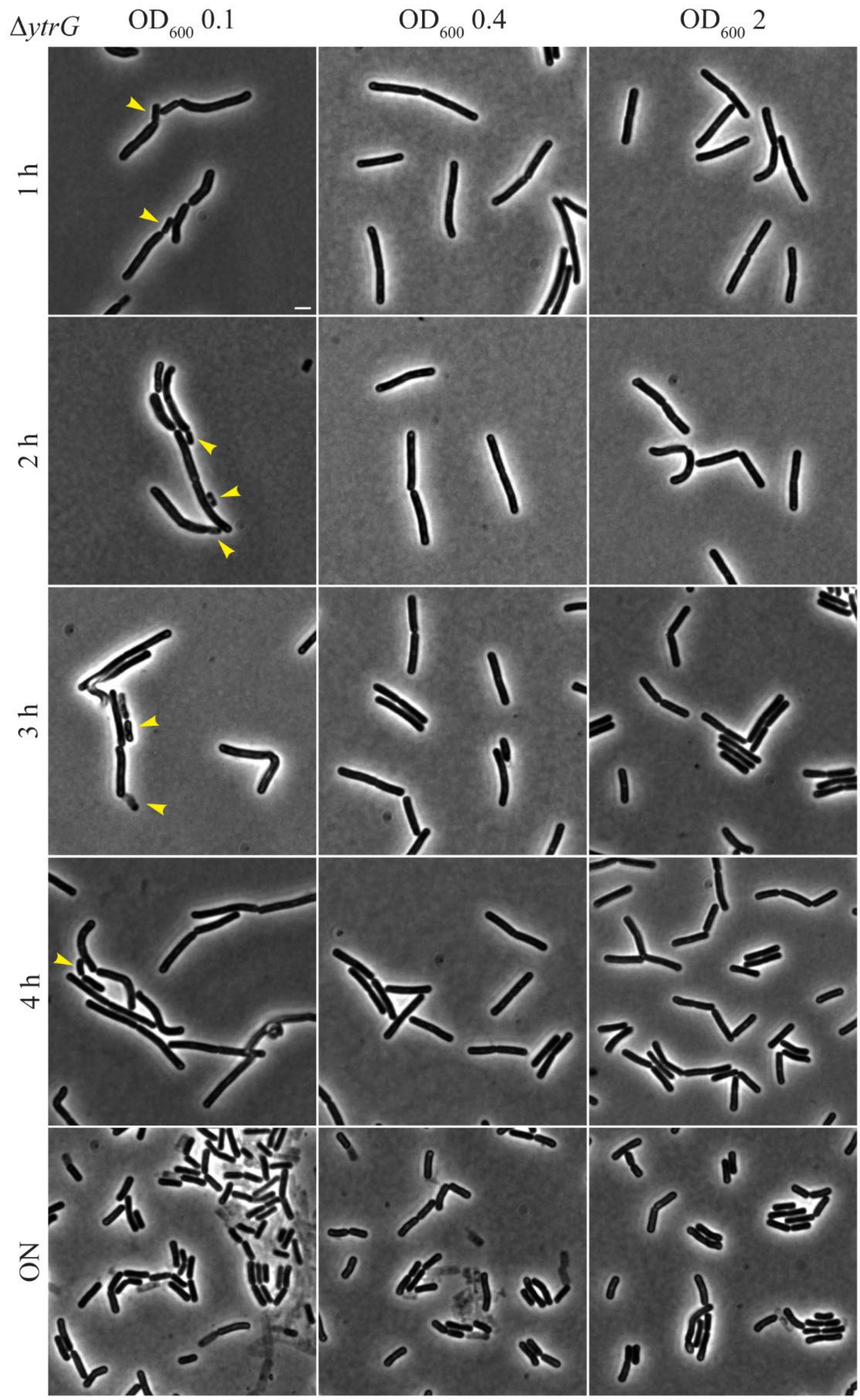

Figure S9: Phase contrast microscopy of *B. subtilis* GP2699 (Δ*ytrG*) subjected to a temperature shift from 37 to 24 °C at different ODs. Scale bar 2 µm. Yellow arrows indicate small cells.

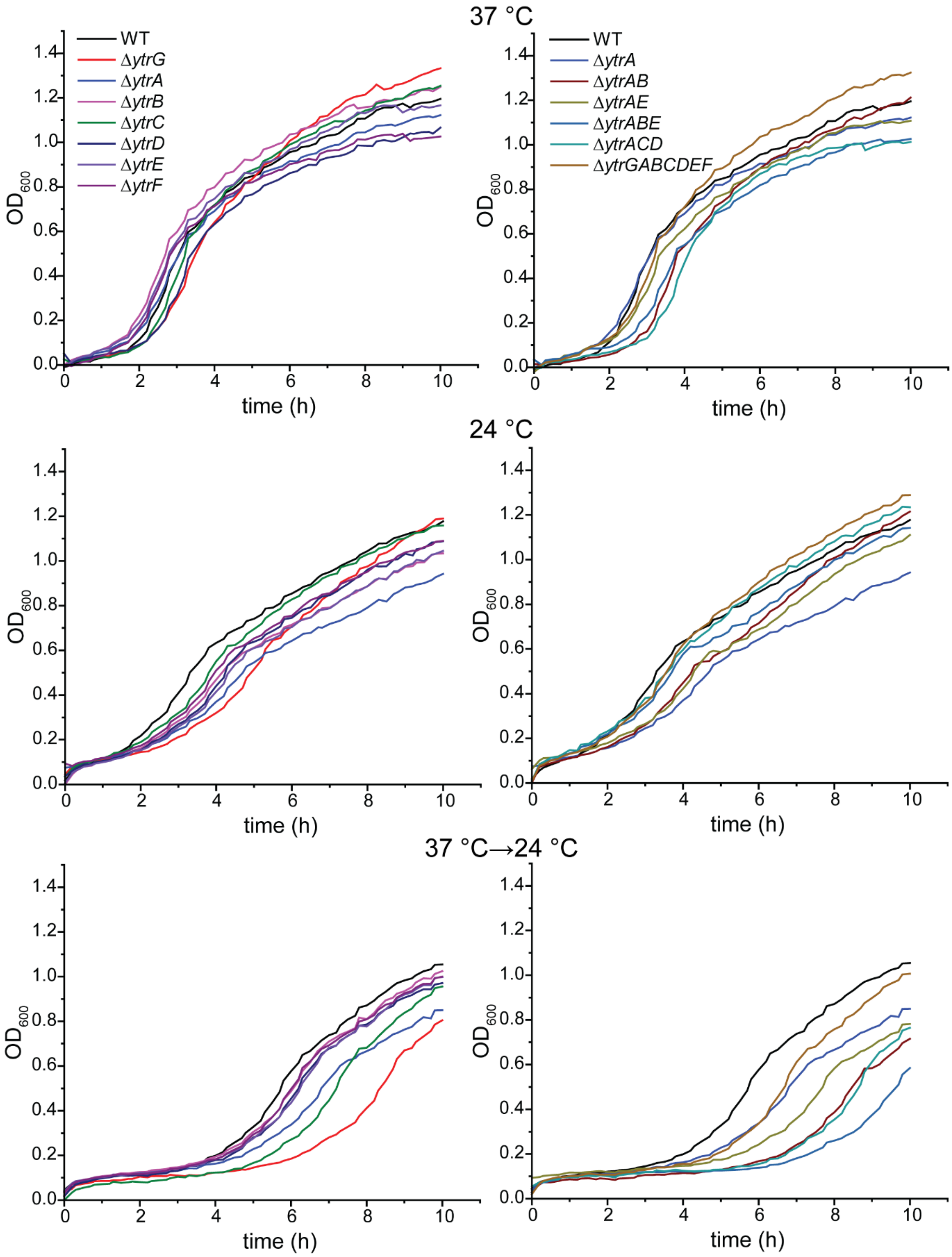

Figure S10: Growth curves of *B. subtilis* 168CA (WT) and *ytr* mutant strains under different temperature conditions. Cultures were inoculated from stationary overnight cultures.

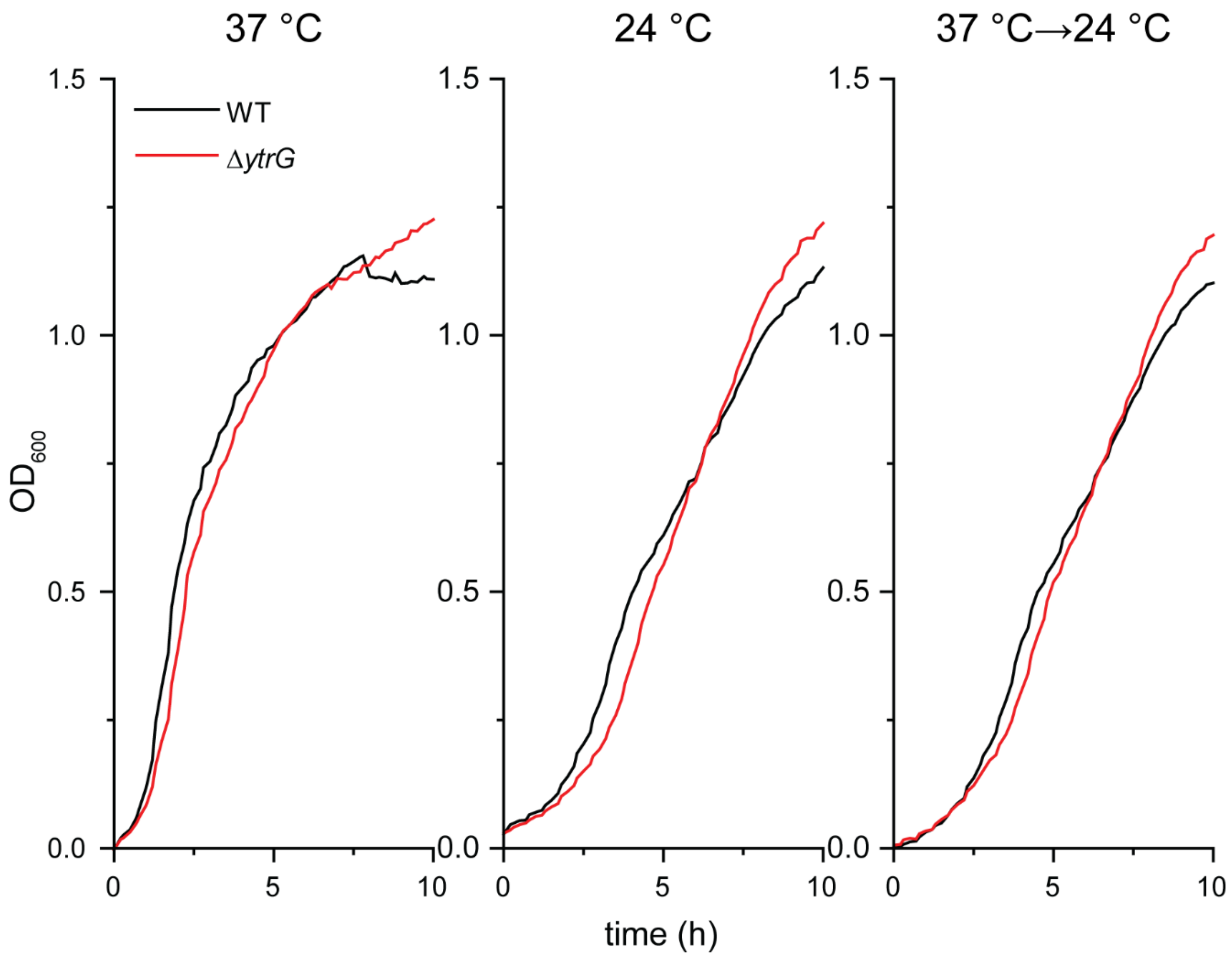

Figure S11: Growth curves of *B. subtilis* 168CA (WT) and GP2966 (Δ*ytrG*) under different temperature conditions. Cultures were inoculated from exponentially growing cultures.

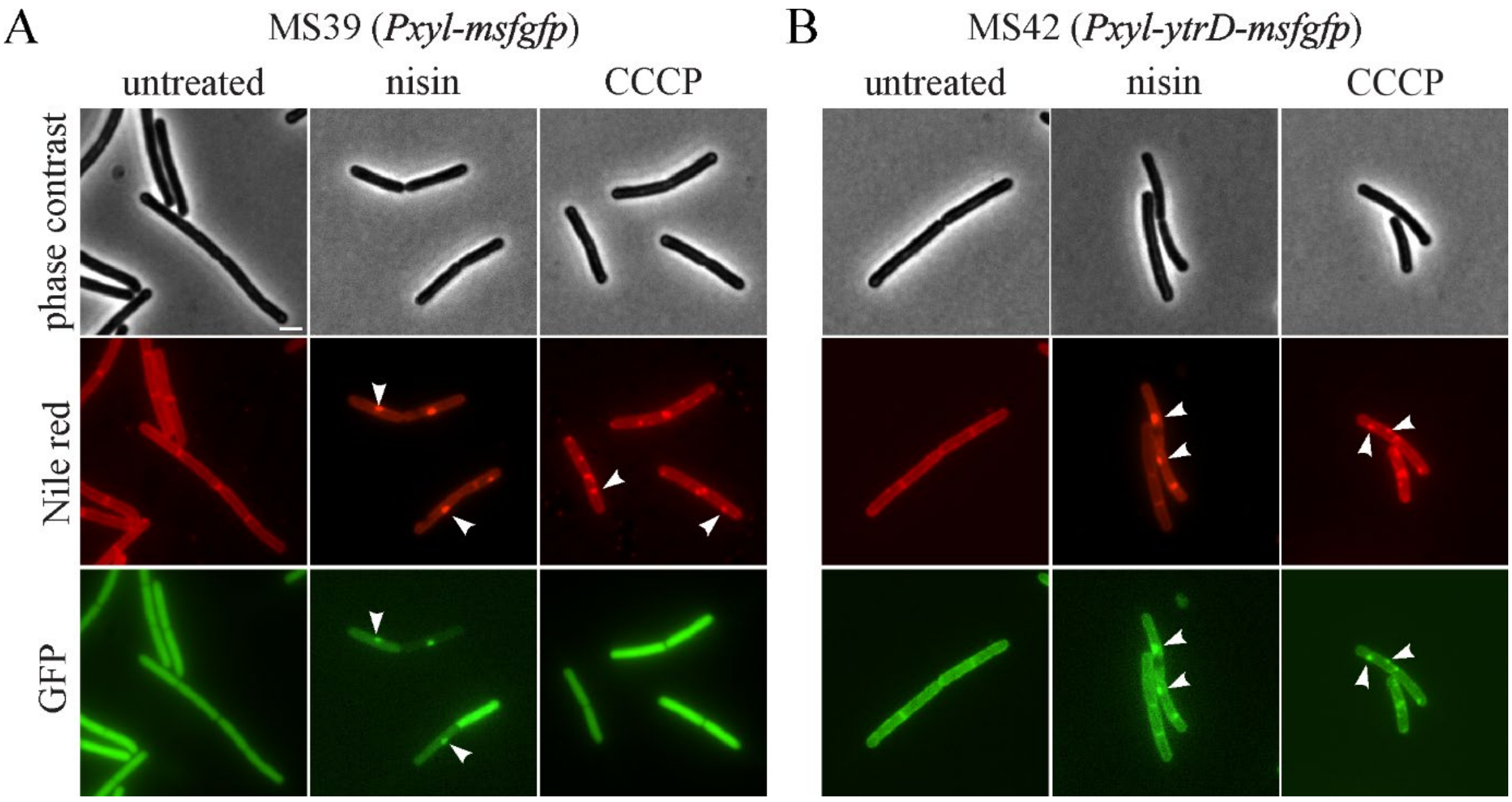

Figure S12: Fluorescence microscopy images of *B. subtilis* MS39 (*Pxyl-msfgfp*) and MS42 (*Pxyl-ytrD-msfgfp*) treated with 0.5 µg/mL nisin and 100 µM CCCP for 10 min. Scale bar 2 µm. Arrows indicate membrane foci and GFP accumulations attracted to antibiotic-induced membrane domains[16,17].

References

(1) Spizizen, J. Transformation of Biochemically Deficient Srains of *Bacillus subtilis* by Deoxyribonucleate. *Proc. Natl. Acad. Sci.* **1958**, *44* (10), 1072–1078. https://doi.org/10.1073/pnas.44.10.1072.

(2) Benda, M.; Schulz, L. M.; Stülke, J.; Rismondo, J. Influence of the ABC Transporter YtrBCDEF of *Bacillus subtilis* on Competence, Biofilm Formation and Cell Wall Thickness. *Front. Microbiol.* **2021**, *12*, 587035. https://doi.org/10.3389/fmicb.2021.587035.

(3) Salzberg, L. I.; Luo, Y.; Hachmann, A.-B.; Mascher, T.; Helmann, J. D. The *Bacillus subtilis* GntR Family Repressor YtrA Responds to Cell Wall Antibiotics. *J. Bacteriol.* **2011**, *193* (20), 5793–5801. https://doi.org/10.1128/JB.05862-11.

(4) Brehm, S. P.; Staal, S. P.; Hoch, J. A. Phenotypes of Pleiotropic-Negative Sporulation Mutants of Bacillus *subtilis*. *J. Bacteriol.* **1973**, *115* (3), 1063–1070. https://doi.org/10.1128/jb.115.3.1063-1070.1973.

(5) Dean, D. R.; Hoch, J. A.; Aronson, A. I. Alteration of the Bacillus *subtilis* Glutamine Synthetase Results in Overproduction of the Enzyme. *J. Bacteriol.* **1977**, *131* (3), 981–987. https://doi.org/10.1128/jb.131.3.981-987.1977.

(6) Srivatsan, A.; Han, Y.; Peng, J.; Tehranchi, A. K.; Gibbs, R.; Wang, J. D.; Chen, R. High-Precision, Whole-Genome Sequencing of Laboratory Strains Facilitates Genetic Studies. *PLoS Genet.* **2008**, *4* (8), e1000139. https://doi.org/10.1371/journal.pgen.1000139.

(7) Hoffmann, T.; Boiangiu, C.; Moses, S.; Bremer, E. Responses of Bacillus *subtilis* to Hypotonic Challenges: Physiological Contributions of Mechanosensitive Channels to Cellular Survival. *Appl. Environ. Microbiol.* **2008**, *74* (8), 2454–2460. https://doi.org/10.1128/AEM.01573-07.

(8) Baruah, L.; Sidarta, M.; Hammer úr Skúoy, P.; Johnsson, O.; Frisk, E.; Didelot, P.; Arshad, A.; Wenzel, M. Contribution of the *Bacillus subtilis YtrGABCDEF* Operon to Antibiotic Stress Adaptation. *Microbiol. Spectr.* **2026**, *14* (1), e0269425. https://doi.org/10.1128/spectrum.02694-25.

(9) Gamba, P.; Veening, J. W.; Saunders, N. J.; Hamoen, L. W.; Daniel, R. A. Two-Step Assembly Dynamics of the *Bacillus subtilis* Divisome. *J. Bacteriol.* **2009**, *191* (13), 4186–4194. https://doi.org/10.1128/JB.01758-08.

(10) Marston, A. L.; Thomaides, H. B.; Edwards, D. H.; Sharpe, M. E.; Errington, J. Polar Localization of the MinD Protein of Bacillus *subtilis* and Its Role in Selection of the Mid-Cell Division Site. *Genes Dev.* **1998**, *12* (21), 3419–3430. https://doi.org/10.1101/gad.12.21.3419.

(11) Bachmann, B. J. Derivations and Genotypes of Some Mutant Derivatives of *Escherichia Coli* K-12. In *Escherichia coli and Salmonella: cellular and molecular biology*; Neidhardt, F.C.; Ingraham, J.L.; Low, K.B.; Magasanik, B.; Schaechter, M.; Umbarger, H. E., Ed.; American Society for Microbiology: Washington, D.C., 1987; pp 1190–1219.

(12) Kloos, W. E.; Ballard, D. N.; George, C. G.; Webster, J. A.; Hubner, R. J.; Ludwig, W.; Schleifer, K. H.; Fiedler, F.; Schubert, K. Delimiting the Genus *Staphylococcus* through Description of Macrococcus *caseolyticus* Gen. Nov., Comb. Nov. and Macrococcus *equipercicus* Sp. Nov., Macrococcus *bovicus* Sp. Nov. and Macrococcus *carouselicus* Sp. Nov. *Int. J. Syst. Bacteriol.* **1998**, *48* (3), 859–877. https://doi.org/10.1099/00207713-48-3-859.

(13) Müller, A.; Wenzel, M.; Strahl, H.; Grein, F.; Saaki, T. N. V.; Kohl, B.; Siersma, T.; Bandow, J. E.; Sahl, H.-G.; Schneider, T.; Hamoen, L. W. Daptomycin Inhibits Cell Envelope Synthesis by Interfering with Fluid Membrane Microdomains. *Proc. Natl.*

Acad. Sci. **2016**, *113* (45). https://doi.org/10.1073/pnas.1611173113.
(14) Jones, P.; Binns, D.; Chang, H.-Y.; Fraser, M.; Li, W.; McAnulla, C.; McWilliam, H.; Maslen, J.; Mitchell, A.; Nuka, G.; Pesseat, S.; Quinn, A. F.; Sangrador-Vegas, A.; Scheremetjew, M.; Yong, S.-Y.; Lopez, R.; Hunter, S. InterProScan 5: Genome-Scale Protein Function Classification. *Bioinformatics* **2014**, *30* (9), 1236–1240. https://doi.org/10.1093/bioinformatics/btu031.
(15) Gautier, R.; Douguet, D.; Antonny, B.; Drin, G. HELIQUEST: A Web Server to Screen Sequences with Specific Alpha-Helical Properties. *Bioinformatics* **2008**, *24* (18), 2101–2102. https://doi.org/10.1093/bioinformatics/btn392.
(16) Schäfer, A.-B.; Sidarta, M.; Abdelmesseh Nekhala, I.; Marinho Righetto, G.; Arshad, A.; Wenzel, M. Dissecting Antibiotic Effects on the Cell Envelope Using Bacterial Cytological Profiling: A Phenotypic Analysis Starter Kit. *Microbiol. Spectr.* **2024**, *12* (3), 1–18. https://doi.org/10.1128/SPECTRUM.03275-23/FORMAT/EPUB.
(17) Strahl, H.; Burmann, F.; Hamoen, L. W. The Actin Homologue MreB Organizes the Bacterial Cell Membrane. *Nat. Commun.* **2014**, *5*, 3442. https://doi.org/10.1038/ncomms4442.
